# Supplementary material for: Diel Variations of Extracellular Microcystin Influence the Subcellular Dynamics of RubisCO in Microcystis aeruginosa PCC 7806
Source: Microorganisms. 2021 Jun 10;9(6):1265. doi: 10.3390/microorganisms9061265 (PMC8230624; doi:10.3390/microorganisms9061265)
Supplement: Supplementary file 1 [file microorganisms-09-01265-s001.zip › microorganisms-1235773-supplementary.pdf]

microorganisms

Supplementary materials

Table S1. Primers used in qRT-PCR.

Target Gene	5′–3′-Sequence
IPF47 (forward)	AAACTGCCGGTCAAAATCCC
IPF47 (reverse)	GCCGCTCAAAATTGCTTTCC
mcyA (forward)	TGGGTTGATAGGGTGCCAAT
mcyA (reverse)	CCTCAATCTCAGCAAGGGGA
rbcL (forward)	AATCAAAACCCCCATCATCA
rbcL (reverse)	TGATCACCACCAGAGAGACG
rbcS (forward)	CTCACCGATCAACAAATTGC
rbcS (reverse)	TTCTTGGGGACCAGAAACAG
rnpB (forward)	GGGGTAAGGGTGCAAAGGT
rnpB (reverse)	AGACCAACCTTTGTCCCTCC

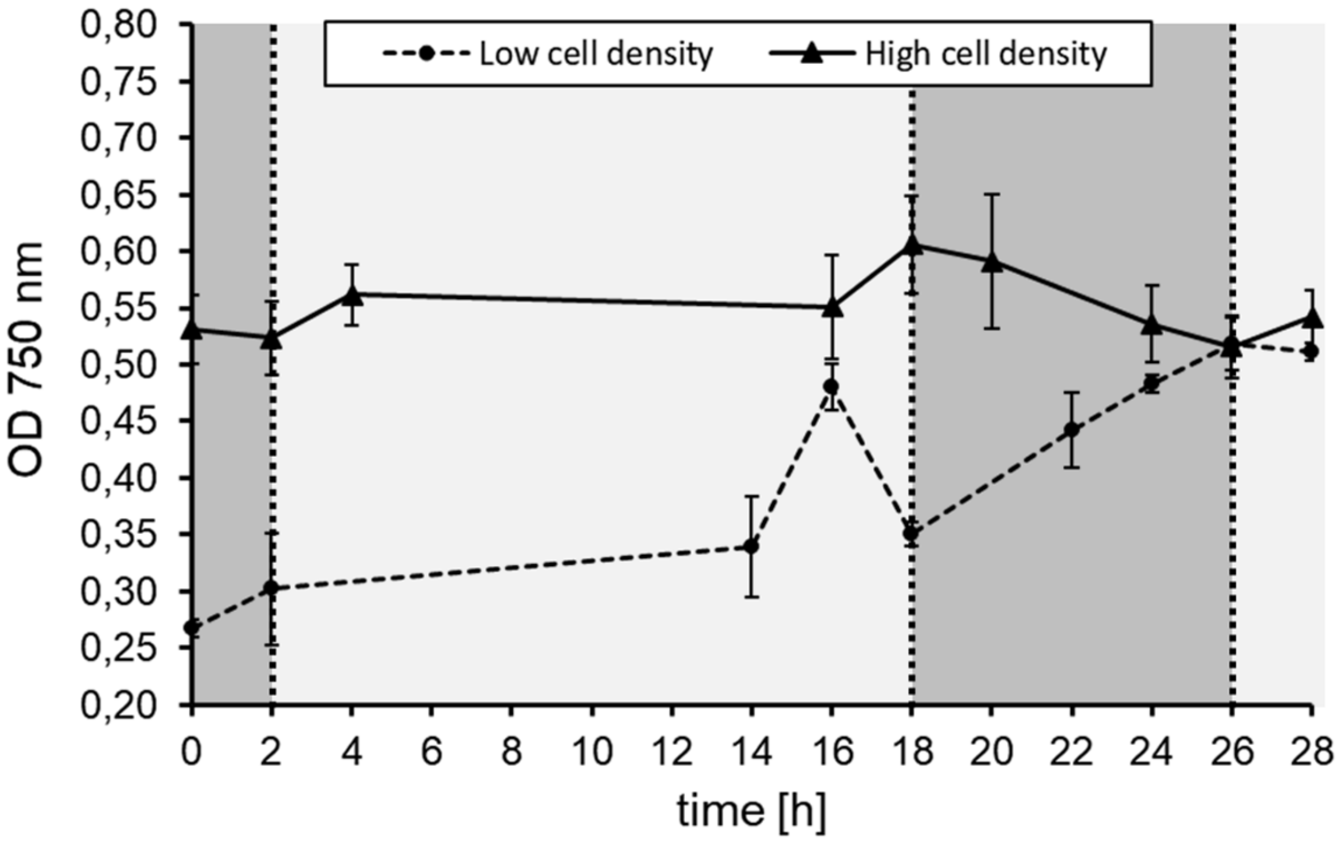

Figure S1. Growth curves of liquid cultures of Microcystis during a diel growth experiment. Three biological replicates of liquid cultures at two different initial cell densities were sampled a 9 time points and the optical densities were recorded photometrically at 750 nm. Light absorption properties of Microcystis cells can vary considerably with fluctuations in gas vesicle content, leading to frequent outlier data points. Lighter and darker shading corresponds to day and night phases, respectively.

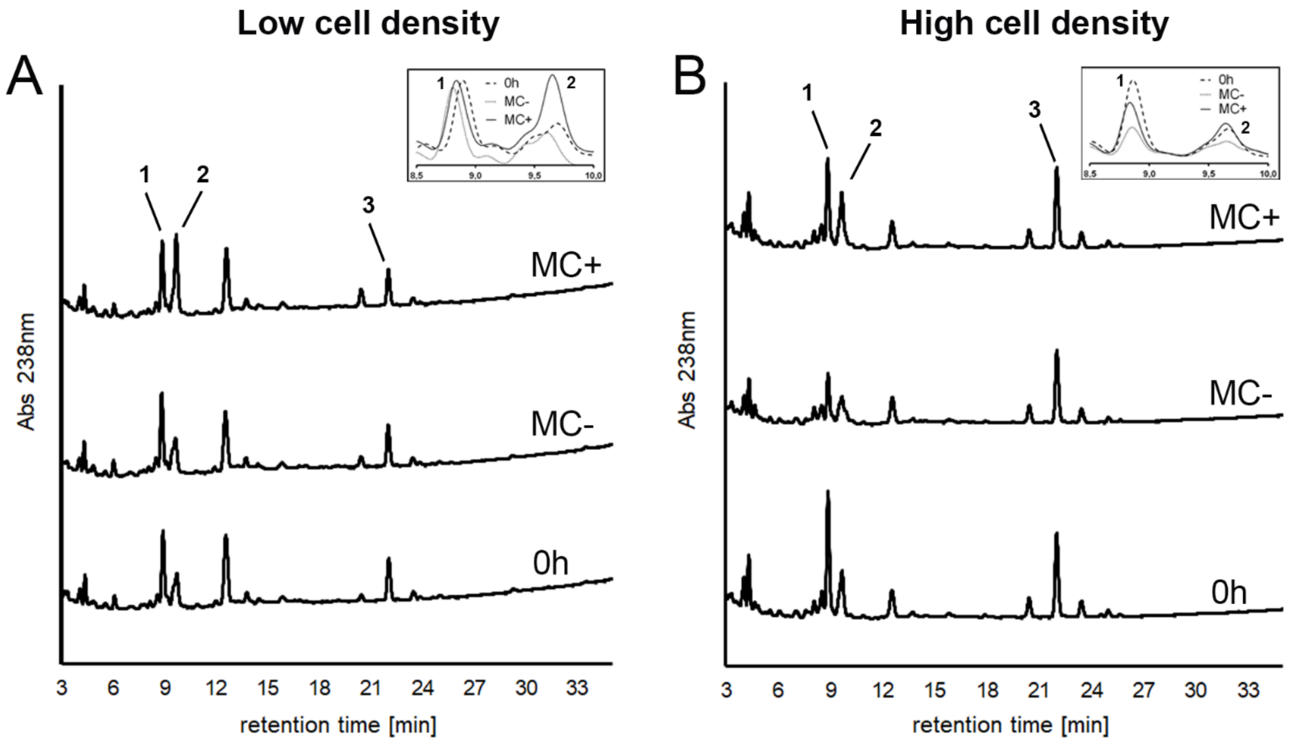

Figure S2. HPLC profiles of culture supernatants of liquid cultures of Microcystis from a microcystin-addition experiment. Liquid cultures at low (A) and high (B) cell densities were grown for 2 hours at high light with the addition of MC to the growth medium (MC+) or without it (MC-). Culture supernatants were analyzed by HPLC. Insets show magnifications of the two main MC variant peaks from the same chromatograms.

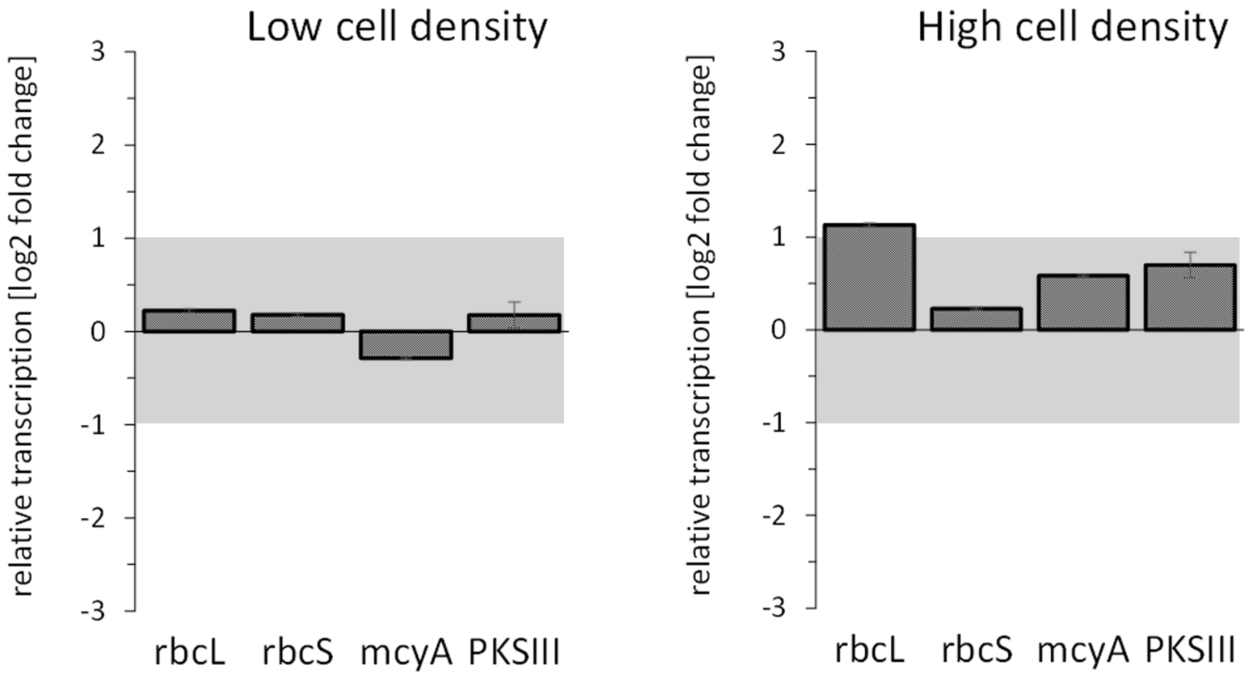

Figure S3. Effects on transcription of microcystin addition to Microcystis liquid cultures. Low-light adapted liquid cultures at low and high cell densities were exposed for 2 hours to high light with or without the addition of MC to the growth medium. Total RNA was isolated and relative transcription of four genes analyzed by qRT-PCR. Values are normalized to samples without MC addition (log2 fold change = 0). Grey shading indicates interval of log2 fold changes between -1 and 1. Measurements were performed in triplicates.
